# How Is Generative AI Use Associated with Employee Creative Behavior? Uncovering Capability- and Risk-Based Psychological Pathways

**DOI:** 10.3390/bs16071254

**Published:** 2026-07-22

**Authors:** Jingya Yang, Haikun Shan, Ji-Na Lee, Zhaoqi Li

**Affiliations:** 1 Department of Global Management, Seokyeong University, Seoul 02713, Republic of Korea; 2 Department of Business, Gachon University, Seongnam 13120, Republic of Korea

**Keywords:** generative AI use, employee creative behavior, creative self-efficacy, psychological safety, digital leadership

## Abstract

The rapid diffusion of generative AI is profoundly transforming knowledge-intensive work and reshaping how employees innovate. Although prior research has highlighted the positive role of generative AI in improving work efficiency and performance, the underlying mechanisms through which it promotes employee creative behavior remain insufficiently understood. Drawing on Social Cognitive Theory and Psychological Safety Theory, this study develops a moderated dual-path model to examine how generative AI use influences employee creative behavior through creative self-efficacy and psychological safety. Using a three-wave time-lagged survey design, data were collected from 297 employees in highly digitalized industries. The results indicate that generative AI use is positively associated with subsequent self-reported employee creative behavior. Both creative self-efficacy and psychological safety significantly mediate this relationship. Furthermore, digital leadership strengthens the positive effects of generative AI use on creative self-efficacy and psychological safety, thereby further enhancing its indirect effects on employee creative behavior. The findings not only extend the literature on generative AI and employee creative behavior but also deepen the theoretical understanding of AI empowerment mechanisms by identifying two critical pathways: a capability-enhancement pathway and a risk-reduction pathway. This study provides important theoretical insights and practical implications for organizations seeking to effectively leverage generative AI to foster employee creative behavior in the context of digital transformation.

## 1. Introduction

The rapid advancement of Artificial Intelligence (AI) and Generative AI is increasingly reshaping workplace practices, knowledge-intensive work, and interactions between employees and technology (Braganza et al., 2021; Brynjolfsson et al., 2025; Chen & Li, 2026; Huang & Rust, 2018; Li et al., 2025). Although existing studies have examined the role of generative AI in enhancing knowledge-worker productivity, task quality, and operational efficiency (Dell’Acqua et al., 2023; Noy & Zhang, 2023; Xia et al., 2026), its relationship with employee creative behavior—an important driver of organizational innovation—remains insufficiently understood (Jia et al., 2024; Wang et al., 2026). In this study, employee creative behavior refers to the generation and implementation of novel and useful ideas within organizational contexts (Amabile, 1996; Anderson et al., 2014). Prior research on AI and creativity has primarily focused on the broader effects of technology, creative outputs, or innovation outcomes (Ding et al., 2026; Doshi & Hauser, 2024), leaving a significant gap in understanding how employees’ actual use of generative AI is associated with individual-level creative behavior in the workplace.

The relationship between generative AI use and employee creative behavior should not be understood as a simple “tool use–behavioral outcome” process. Generative AI may provide employees with informational support, alternative perspectives, and feedback, but the availability of these resources does not automatically correspond to higher levels of employee creative behavior.

Rather, their value depends on how employees interpret AI-assisted work experiences, develop confidence in their creative capabilities, and perceive the interpersonal risks of expressing AI-supported ideas in the workplace (Bankins et al., 2024; Raisch & Krakowski, 2021; Vial, 2019). When generative AI becomes embedded in daily work processes, it is associated not only with employees’ access to resources but also shapes how they evaluate their creative capabilities, express uncertain ideas, and manage the risks associated with trying new approaches (Wang et al., 2026). The effectiveness of generative AI therefore depends not only on its technical capabilities but also on how employees interpret, utilize, and integrate AI-generated information and knowledge within organizational contexts. In highly digitalized and AI-supported work contexts, it remains unclear whether and how AI-supported information, suggestions, and feedback are associated with creative confidence, perceived interpersonal safety, and ultimately creative behavior.

Moreover, the creative value of generative AI does not automatically emerge simply because employees use AI tools. Although generative AI can provide rapid feedback, alternative suggestions, and creative support, its outputs are not always accurate, reliable, or highly original (Pan et al., 2026). AI-generated content may contain informational biases, logical errors, or homogenized perspectives (Bankins et al., 2024; Dwivedi et al., 2023). Therefore, when using generative AI, employees need not only to access the informational support provided by AI, but also to possess the ability to critically evaluate AI-generated outputs, recognize their limitations, and integrate them with their own professional judgment (Glikson & Woolley, 2020). Prior research emphasizes that effective AI-assisted work requires users to maintain critical judgment, calibrate their reliance on AI, and integrate AI-generated suggestions with their own professional expertise (Glikson & Woolley, 2020; Jarrahi, 2018). Without effective judgment and oversight, employees may become overly reliant on AI-generated suggestions, potentially weakening independent thinking and even leading to the homogenization of creative outcomes (Dwivedi et al., 2023). Therefore, the role of generative AI in creative work should be understood as the result of the joint influence of human–AI collaboration and employees’ cognitive judgment, rather than being determined solely by the use of the technology itself (Jarrahi, 2018; Raisch & Krakowski, 2021).

To explain how generative AI use is associated with employee creative behavior, this study proposes two complementary psychological pathways: creative self-efficacy and psychological safety. Although both mechanisms may be influenced by generative AI use, they represent distinct psychological processes underlying creative behavior, namely capability perception and risk perception.

First, generative AI use may be associated with employee creative behavior through creative self-efficacy. Creative self-efficacy refers to an individual’s belief in their ability to produce creative outcomes (Tierney & Farmer, 2002). According to Social Cognitive Theory, self-efficacy is developed through mastery experiences, feedback, observational learning, and external informational support (Bandura, 1986, 1997). Unlike traditional digital tools, generative AI not only provides information but also generates alternative solutions, refines creative content, and offers immediate feedback, thereby assisting employees in performing creative tasks more effectively. In AI-supported work environments, employees can more easily obtain creative inspiration, improve problem-solving approaches, and reduce cognitive uncertainty during the creative process. As a result, they are more likely to develop positive beliefs about their creative capabilities. Because creative self-efficacy enhances persistence, exploration, and cognitive engagement in creative tasks, it may serve as an important capability-based pathway linking generative AI use to employee creative behavior. At the same time, generative AI use may also promote employee creative behavior by enhancing psychological safety. Psychological safety refers to employees’ perceptions that they can express ideas, raise questions, and experiment with new approaches without fear of negative evaluation, interpersonal rejection, or adverse consequences (Edmondson, 1999). According to Psychological Safety Theory, when employees perceive lower interpersonal risk, they are more willing to voice opinions, share knowledge, and experiment with new ways of working, thereby facilitating innovation and creative behavior (Frazier et al., 2017). Compared with traditional digital tools, generative AI can provide creative support, alternative solutions, and real-time feedback throughout the creative process, helping employees reduce uncertainty associated with innovation attempts. By using AI to rehearse, refine, and improve their ideas before sharing them with others, employees may perceive the risks of failure and negative social evaluation as more manageable, thereby reducing concerns about expressing imperfect or unconventional ideas. In this sense, generative AI functions not only as a knowledge-support tool but also as a risk-buffering mechanism that enhances psychological safety. As perceived interpersonal risks decrease, employees become more likely to express novel ideas, engage in creative discussions, and experiment with new work methods, which is subsequently associated with higher levels of creative behavior.

Leadership approaches can influence employees’ behavior by shaping their psychological perceptions, and these effects may be contingent on the work context (Li & Jo, 2025). Digital leadership refers to leaders’ ability to provide digital vision, technological support, resource coordination, and transformational guidance in digital work environments (Avolio et al., 2014; Zeike et al., 2019). Leaders with strong digital leadership are more likely to clarify the value of digital technologies, provide training and resource support, and encourage employees to experiment with new digital work practices (Klus & Müller, 2021; Zeike et al., 2019; Zhu et al., 2022). Under such leadership conditions, employees are more likely to perceive generative AI as a source of capability development and creative support rather than as an ambiguous or potentially risky technology. In contrast, when digital leadership is weak, employees may lack the guidance and support necessary to effectively integrate generative AI into creative work. Therefore, digital leadership may strengthen the positive effects of generative AI use on both creative self-efficacy and psychological safety.

Although recent studies have begun to examine the relationship between generative AI use and employee creative behavior (Jia et al., 2024), several important theoretical gaps remain. First, although prior research has proposed dual-pathway mechanisms linking generative AI use to employee creativity (Wang et al., 2026), existing studies have largely focused on mediators related to creative process engagement, emotional experiences, or general resource empowerment, while paying insufficient attention to how generative AI simultaneously shapes employees’ capability perception and risk perception. In other words, current research has not fully explained how generative AI use may both enhance employees’ confidence in their creative abilities and reduce the interpersonal risks they perceive when expressing novel ideas. Second, existing research has paid limited attention to the contextual dependence of generative AI’s effects. The effectiveness of generative AI is not determined solely by task characteristics or individual employee attributes; rather, its impact is also highly contingent on the organizational support environment, particularly the role of leadership in providing technological support, resource coordination, and innovation guidance (Zhu et al., 2022).

Therefore, drawing on Social Cognitive Theory and Psychological Safety Theory, this study develops a moderated dual-path mediation model to examine how generative AI use is associated with employee creative behavior through creative self-efficacy and psychological safety, while further investigating the boundary role of digital leadership. To test the proposed hypotheses, a three-wave time-lagged survey design was employed, and data were collected from 297 employees working in digitalized enterprises in China. Specifically, this study seeks to address the following research questions: Is generative AI use positively associated with employee creative behavior? Do creative self-efficacy and psychological safety explain this relationship? Does digital leadership strengthen these pathways? By addressing these questions, this study aims to uncover the capability- and risk-based psychological pathways linking generative AI use to employee creative behavior, thereby enriching the theoretical understanding of employee creative behavior in the context of digital transformation.

## 2. Theoretical Background and Hypotheses

### 2.1. Social Cognitive Theory and Psychological Safety Theory

This study draws on Social Cognitive Theory and Psychological Safety Theory to explain how generative AI use is associated with employee creative behavior.

Social Cognitive Theory suggests that human behavior is shaped by the reciprocal interaction among personal cognition, environmental factors, and behavioral experiences (Bandura, 1986). One of its core concepts is self-efficacy, which refers to an individual’s belief in their ability to successfully perform a specific task (Bandura, 1997). In creative work contexts, creative self-efficacy reflects employees’ confidence in their ability to generate novel and useful ideas (Edmondson & Lei, 2014; Tierney & Farmer, 2002). Because creative behavior often requires exploration, sustained effort, and the ability to cope with uncertainty (Oldham & Cummings, 1996; Shalley et al., 2004; Woodman et al., 1993), the examples, alternative solutions, preliminary ideas, and immediate feedback provided by generative AI can reduce the difficulty of open-ended creative tasks and strengthen employees’ confidence in completing creative work (Garbuio & Lin, 2021). As employees accumulate AI-supported experiences, they may gradually develop stronger creative self-efficacy, which in turn promotes creative behavior (Tierney & Farmer, 2011).

Psychological Safety Theory, in contrast, emphasizes that perceptions of interpersonal risk influence whether employees are willing to express opinions, raise questions, and experiment with new approaches (Edmondson, 1999; Frazier et al., 2017). Creative work is often associated with high levels of uncertainty and risk, requiring employees to share incomplete ideas, challenge established routines, and face the possibility of criticism or rejection (Carmeli et al., 2009, 2010). Unlike traditional digital tools, generative AI not only provides information support but also helps employees organize complex knowledge, refine their logic, and improve the presentation of their ideas, thereby reducing cognitive strain and communication uncertainty during creative expression. As a result, employees who use generative AI may feel more comfortable expressing novel ideas, engaging in creative discussions, and experimenting with new ways of working, thereby experiencing higher levels of psychological safety (Edmondson & Lei, 2014). Psychological safety may therefore represent an important social pathway linking generative AI use to employee creative behavior.

Furthermore, the effectiveness of these psychological pathways may depend on leadership context. Digital leadership refers to leaders’ ability to provide technological vision, resource support, usage guidance, and tolerance for responsible experimentation in digital work environments (Kane et al., 2019). Such leadership helps employees understand the value and boundaries of generative AI, reduces uncertainty associated with AI use, and fosters a work environment that supports creative exploration (Klus & Müller, 2021). Consequently, digital leadership may strengthen the positive effects of generative AI use on both creative self-efficacy and psychological safety.

In summary, Social Cognitive Theory and Psychological Safety Theory provide important theoretical foundations for understanding how generative AI use is associated with employee creative behavior. The former explains how AI-supported experiences enhance employees’ confidence in their creative capabilities, whereas the latter highlights how AI-assisted work environments reduce interpersonal risks associated with creative interactions. Although both creative self-efficacy and psychological safety explain the relationship between generative AI use and employee creative behavior, they represent distinct psychological mechanisms: creative capability perception and interpersonal risk perception. Specifically, creative self-efficacy concerns whether employees believe they are capable of generating and implementing creative ideas, whereas psychological safety concerns whether employees believe it is socially safe to express, discuss, and refine those ideas in the workplace. By simultaneously examining these two pathways, this study offers a more comprehensive understanding of how generative AI use is associated with employee creative behavior in the workplace.

### 2.2. Generative AI Use and Employee Creative Behavior

Generative AI use refers to the extent to which employees utilize generative artificial intelligence tools in their work activities, including information searching, content generation, idea development, problem solving, and knowledge integration. Unlike traditional digital tools that primarily function as information repositories or task automation systems, generative AI can proactively provide suggestions, generate alternative solutions, offer illustrative examples, and deliver real-time feedback. As a result, it plays a more active role in open-ended and knowledge-intensive tasks and serves as a cognitive support tool for creative work (Füller et al., 2022; Mariani & Dwivedi, 2024; Verganti et al., 2020). Generative AI use may be positively associated with employee creative behavior because it provides employees with informational support, alternative perspectives, real-time feedback, and opportunities for idea refinement. These resources can help employees identify problems, generate novel solutions, improve preliminary ideas, and implement creative outputs in their work. Therefore, employees who use generative AI more actively are more likely to engage in creative behavior.

**Hypothesis** **1.** *Generative AI use is positively associated with employee creative behavior.*

### 2.3. The Mediating Role of Creative Self-Efficacy

According to SCT, individuals develop perceptions and judgments about their capabilities through mastery experiences, feedback, observational learning, and interactions with their environment (Bandura, 1986, 1997). In the context of generative AI use, employees can continuously access informational support, task recommendations, alternative perspectives, and feedback resources. These resources not only improve the efficiency of completing complex tasks but also reduce the cognitive burden associated with creative work. As employees repeatedly experience successful task completion and creative progress with the assistance of generative AI, their perceptions of competence and control are likely to increase.

Such capability beliefs are primarily reflected in creative self-efficacy, which refers to an individual’s belief in their ability to generate novel and useful ideas (Tierney & Farmer, 2002). Because generative AI is widely used for idea generation, content refinement, and innovative problem solving, the positive experiences gained through AI-supported work are more likely to strengthen employees’ confidence in their creative abilities. As these successful experiences accumulate, employees become increasingly confident in their capacity to generate, improve, and implement innovative ideas.

Moreover, generative AI can expand employees’ awareness of potential innovation opportunities and solution pathways by providing diverse perspectives, preliminary solutions, and reference examples. Creative work often involves incomplete information and ambiguous problems. In such situations, generative AI can participate in the creative process by generating novel suggestions, simulating alternative viewpoints, and offering iterative feedback. This co-creation characteristic enables employees to continuously experience creative success and strengthens their confidence in their creative potential, thereby encouraging greater engagement in innovative activities.

Creative self-efficacy is an important psychological resource that promotes creative behavior. Employees with higher levels of creative self-efficacy are more likely to persist in exploring new ideas, take creative risks, and invest sustained effort in developing innovative solutions (Garbuio & Lin, 2021; Tierney & Farmer, 2002). Prior research has consistently shown that creative self-efficacy helps explain how individual experiences and contextual resources are translated into creativity and innovative behavior (Gong et al., 2009; Hülsheger et al., 2009). Therefore, when generative AI use provides employees with continuous task support, learning opportunities, and successful creative experiences, their creative self-efficacy is likely to increase, which in turn facilitates creative behavior.

From the perspective of Social Cognitive Theory, generative AI use may provide employees with mastery experiences, feedback, and informational support during creative tasks. These AI-assisted experiences can strengthen employees’ beliefs that they are capable of generating, refining, and implementing novel ideas. Therefore, creative self-efficacy serves as a psychological mechanism linking generative AI use to employee creative behavior. Specifically, generative AI use can strengthen employees’ confidence in their creative capabilities by providing informational support, alternative solutions, and opportunities for idea refinement. Through repeated experiences of AI-assisted problem solving and creative success, employees gradually develop stronger beliefs in their ability to generate and implement innovative ideas. These enhanced efficacy beliefs further motivate employees to engage actively in creative activities, persist in the face of challenges, and invest greater effort in developing innovative solutions.

This study focuses on creative self-efficacy as an important psychological mechanism through which generative AI use is associated with employee creative behavior. Rather than assuming that AI use automatically produces creative outcomes, we argue that employees’ confidence in their creative capability helps explain this relationship. In other words, creative self-efficacy serves as an important psychological mechanism through which generative AI use is associated with employee creative behavior.

Based on the above reasoning, the following hypothesis is proposed:

**Hypothesis** **2.** *Creative self-efficacy mediates the relationship between generative AI use and employee creative behavior.*

### 2.4. The Mediating Role of Psychological Safety

Psychological safety refers to employees’ perceptions that they can express ideas, ask questions, acknowledge uncertainty, and experiment with new approaches without fear of negative interpersonal consequences (Edmondson, 1999; Frazier et al., 2017). Although the concept was originally developed in team-based contexts, it can also be understood at the individual level, reflecting employees’ subjective perceptions of interpersonal risk in the workplace. In this study, psychological safety refers to employees’ perceived sense of safety when expressing ideas, trying new approaches, and discussing uncertain work-related issues during creative tasks.

Psychological safety is a critical antecedent of creative behavior because creative activities inherently involve uncertainty, risk of failure, and interpersonal exposure. Employees are often required to propose undeveloped ideas, challenge existing work practices, and explore novel solutions, which may expose them to criticism, rejection, or negative evaluations. When employees perceive a high level of psychological safety, they are more willing to speak up, seek feedback, share ideas, and engage in trial-and-error learning processes, thereby facilitating the development and refinement of innovative ideas (Hülsheger et al., 2009; Shalley et al., 2004).

Psychological safety is particularly important in the context of generative AI use. Employees must not only evaluate and revise AI-generated outputs but also decide whether to share AI-assisted ideas and explain how AI has been utilized in their work. If employees fear that using AI may be interpreted as a sign of incompetence, excessive dependence on technology, or a lack of originality, they may avoid openly discussing AI-supported ideas, thereby limiting the creative potential of AI. In contrast, generative AI can enhance psychological safety by providing informational support, alternative perspectives, and preliminary solutions that help employees better understand problems, refine ideas, and optimize decision-making processes. Employees can test, revise, and improve their ideas with AI before presenting them to others, which reduces concerns about failure and negative evaluations.

As employees accumulate experience with generative AI, they become more capable of coping with uncertainty in creative tasks and gain greater confidence in interpersonal interactions. Compared with traditional digital tools, generative AI provides continuous feedback and suggestions, offering employees more opportunities to validate and refine their ideas before expressing them publicly. Consequently, generative AI use can reduce perceived interpersonal risk and enhance employees’ psychological safety.

According to Psychological Safety Theory, employees who perceive lower levels of interpersonal risk are more likely to express opinions, share knowledge, offer suggestions, and engage in innovative activities (Edmondson, 1999; Frazier et al., 2017). Creative behavior is inherently uncertain and risky; therefore, employees’ willingness to express and implement innovative ideas largely depends on their level of psychological safety. Employees with higher psychological safety are more likely to seek feedback, discuss unfinished ideas, challenge existing routines, and take innovation-related risks, all of which facilitate the generation and implementation of creative ideas. Conversely, employees with low psychological safety tend to remain silent, avoid expressing divergent opinions, and refrain from experimenting with new approaches, thereby inhibiting creative behavior. Previous studies have consistently demonstrated that psychological safety promotes employee voice behavior, knowledge sharing, and innovative behavior (Edmondson, 1999; Frazier et al., 2017; Hülsheger et al., 2009). Therefore, when employees experience higher levels of psychological safety, they are more likely to engage actively in the creative process and transform innovative ideas into tangible outcomes.

From the perspective of Psychological Safety Theory, employees are more likely to express novel ideas and experiment with new approaches when they perceive lower interpersonal risk. Generative AI use may help employees test, refine, and improve their ideas before sharing them with others, thereby reducing concerns about failure or negative evaluation. Therefore, psychological safety serves as another psychological mechanism linking generative AI use to employee creative behavior. However, technological resources alone do not automatically translate into creative outcomes. Employees must also possess the social and psychological conditions necessary to express ideas, experiment with new approaches, and take innovation-related risks. Psychological safety represents one of the key mechanisms through which this transformation occurs.

Specifically, generative AI use can reduce employees’ perceived interpersonal risk during creative activities by helping them test ideas, refine solutions, and alleviate concerns about failure. When employees believe they can express opinions, discuss unfinished ideas, and engage in innovative experimentation within a relatively safe environment, their level of psychological safety is likely to increase. In turn, higher psychological safety encourages employees to share ideas, seek feedback, engage in trial-and-error learning, and participate more actively in innovative activities, thereby facilitating creative behavior.

This study also focuses on psychological safety as a risk-related psychological mechanism through which generative AI use is associated with employee creative behavior. By reducing perceived interpersonal risk during idea expression and experimentation, psychological safety helps explain the link between generative AI use and employee creative behavior.

Based on the above reasoning, the following hypothesis is proposed:

**Hypothesis** **3.** *Psychological safety mediates the relationship between generative AI use and employee creative behavior.*

### 2.5. The Moderating Role of Digital Leadership

Digital leadership refers to leaders’ ability to establish a clear digital vision, coordinate technological resources, facilitate digital transformation, and help employees adapt to digital change by providing support, guidance, and resources in digital work environments (Avolio et al., 2014). Compared with traditional leadership approaches, digital leadership emphasizes not only the achievement of organizational goals but also the effective application of digital technologies, the cultivation of a digital culture, and the development of employees’ digital competencies (Zeike et al., 2019). In the context of digital transformation, leaders serve not only as resource allocators and decision-makers but also as promoters of digital innovation and facilitators of technological change. Consequently, digital leadership can significantly influence how employees understand, accept, and utilize digital technologies (Kane et al., 2019; Klus & Müller, 2021).

#### 2.5.1. The Moderating Role of Digital Leadership in the Relationship Between Generative AI Use and Creative Self-Efficacy

In the context of generative AI use, employees often face challenges in effectively leveraging AI to support creative work. Although generative AI can provide informational support, alternative solutions, creative suggestions, and continuous feedback (Füller et al., 2022; Mariani & Dwivedi, 2024; Verganti et al., 2020), whether employees develop positive capability beliefs from these resources largely depends on the support and guidance provided by leaders.

According to Social Cognitive Theory, self-efficacy is shaped not only by direct mastery experiences but also by environmental support and social contextual factors (Bandura, 1986, 1997). When leaders provide a clear digital vision, technical training, and resource support, employees are more likely to understand the value of generative AI and acquire the skills necessary to use AI tools effectively (Avolio et al., 2014; Zeike et al., 2019). Under such conditions, employees can more actively utilize AI to search for information, compare alternative solutions, refine creative ideas, and solve complex problems. These experiences provide opportunities for successful task accomplishment and capability development, thereby strengthening employees’ confidence in their creative abilities.

Furthermore, leaders with strong digital leadership tend to encourage employees to explore emerging technologies, experiment with innovative approaches, and engage in constructive feedback processes (Kane et al., 2019; Klus & Müller, 2021). When employees perceive that their leaders support AI-assisted innovation activities, they are more likely to view generative AI as a valuable resource for capability development rather than as a complex or risky technology. As successful experiences with AI continue to accumulate, employees’ confidence in their ability to generate and implement innovative ideas is likely to increase. In contrast, when digital leadership is weak, employees may lack the guidance and support necessary to fully leverage the benefits of generative AI, limiting the extent to which AI-assisted experiences enhance their creative self-efficacy. Therefore, digital leadership is expected to strengthen the positive relationship between generative AI use and creative self-efficacy.

Based on the above reasoning, the following hypothesis is proposed:

**Hypothesis** **4.** *Digital leadership positively moderates the relationship between generative AI use and creative self-efficacy, such that the positive relationship is stronger when digital leadership is high.*

#### 2.5.2. The Moderating Role of Digital Leadership in the Relationship Between Generative AI Use and Psychological Safety

During creative work processes, employees’ use of generative AI is often accompanied by uncertainty and interpersonal risk. Employees may worry that AI-assisted ideas will not be recognized by others or that using AI may be perceived as a sign of incompetence, lack of originality, or excessive dependence on technology. Consequently, even when generative AI provides substantial creative support, employees may hesitate to fully utilize AI resources because of concerns about potential interpersonal consequences.

According to Psychological Safety Theory, employees’ willingness to express ideas, experiment with new approaches, and take innovation-related risks largely depends on whether they perceive their interpersonal environment as safe (Edmondson, 1999; Frazier et al., 2017). Leaders with strong digital leadership are more likely to establish an open, inclusive, and supportive work climate by clarifying AI usage norms, encouraging innovative experimentation, and tolerating reasonable failures (Klus & Müller, 2021; Zeike et al., 2019). Such leadership practices can reduce employees’ concerns about negative evaluations and interpersonal risks.

When employees perceive that their leaders support AI-assisted exploration and recognize the legitimate use of AI in work processes, they are more likely to view generative AI as an innovation tool that is accepted and encouraged by the organization. Under such conditions, employees become more willing to openly discuss AI-generated ideas, seek feedback, and participate in innovative experimentation, thereby enhancing their psychological safety.

Moreover, digital leaders often facilitate open communication and knowledge sharing within teams, creating opportunities for employees to discuss AI applications and exchange innovative ideas (Larjovuori et al., 2016). Such a supportive environment further reduces interpersonal concerns and strengthens employees’ sense of safety during creative activities. Conversely, when digital leadership is insufficient, employees may perceive AI use as uncertain, unsupported, or risky, thereby weakening the positive psychological outcomes associated with generative AI use.

Prior research has also demonstrated that digital leadership promotes employee creativity and innovation by facilitating adaptive work adjustment and strengthening employees’ alignment with digital transformation initiatives (Zhu et al., 2022). Therefore, digital leadership is expected to strengthen the positive effect of generative AI use on psychological safety.

Based on the above reasoning, the following hypothesis is proposed:

**Hypothesis** **5.** *Digital leadership positively moderates the relationship between generative AI use and psychological safety, such that the positive relationship is stronger when digital leadership is high.*

**Figure 1:**
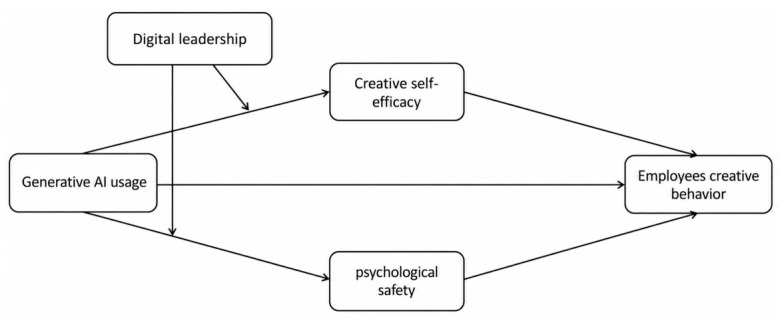
Research model.

Specifically, this study first proposes a positive relationship between generative AI use and employee creative behavior. It then argues that creative self-efficacy and psychological safety serve as two mediating mechanisms: an efficacy-based pathway and a safety-based pathway. Furthermore, digital leadership is proposed as a boundary condition that strengthens the relationships between generative AI use and these two mediators. Accordingly, this study develops a moderated dual-mediation model in which generative AI use is associated with employee creative behavior directly and indirectly through creative self-efficacy and psychological safety, while digital leadership strengthens both first-stage psychological pathways, as illustrated in Figure 1.

## 3. Materials and Methods

### 3.1. Sample and Data Collection

This study employed a three-wave time-lagged survey design to reduce the potential influence of common method bias and enhance temporal separation among the study variables. Given that generative AI is primarily applied in information-intensive and knowledge-based work settings, the target population consisted of employees working in highly digitalized and knowledge-intensive organizations, including those engaged in technical, professional, administrative, analytical, and creative tasks. These employees are more likely to use generative AI tools in their daily work for information processing, idea generation, problem-solving, and other knowledge-intensive activities, making them particularly suitable for the context of this study.

To ensure sample appropriateness, all participants were required to have actual experience using generative AI in their work. Before the formal survey began, a screening question was included to determine whether respondents had previously used generative AI tools for work-related purposes. Individuals without relevant generative AI experience were excluded from the study.

The survey was administered through a professional online survey platform. Prior to participation, all respondents were informed that the study was conducted solely for academic purposes and that their responses would remain anonymous and confidential. Participation was entirely voluntary, and respondents were free to withdraw from the survey at any time without penalty. To minimize social desirability bias and common method variance, participants were informed that there were no “right” or “wrong” answers and were encouraged to respond based on their actual work experiences and perceptions. In addition, the order of questionnaire sections was varied to reduce response pattern bias and consistency effects.

Data were collected in three waves, with a two-week interval between consecutive waves. At Time 1, 500 questionnaires were distributed to employees with experience using generative AI. After excluding incomplete responses and responses that failed to meet the screening criteria, 427 valid questionnaires were retained. The Time 1 survey measured generative AI use, digital leadership, and demographic variables.

Two weeks later, the Time 2 survey was administered to respondents who had completed the Time 1 survey. After excluding unmatched responses and questionnaires with substantial missing data, 352 valid responses were retained, corresponding to a retention rate of 82.4% relative to the valid Time 1 sample. The Time 2 survey measured the two mediating variables: creative self-efficacy and psychological safety.

Following another two-week interval, the Time 3 survey was administered to respondents who had completed the Time 2 survey. After matching responses across all three waves and excluding incomplete or invalid questionnaires, 297 valid matched responses were retained for the final analysis. This represented a retention rate of 69.6% relative to the valid Time 1 sample and a final valid response rate of 59.4% relative to the 500 questionnaires initially distributed. The Time 3 survey measured employee creative behavior.

Several procedures were employed to ensure data quality. First, questionnaires containing substantial missing data were removed. Second, responses exhibiting clear careless response patterns, such as straight-lining behavior or unusually short completion times, were excluded. Third, statistical screening procedures were used to identify potential outliers and multivariate abnormal observations. Specifically, cases with standardized scores exceeding the commonly accepted threshold of |z| > 3.29 were considered univariate outliers. Furthermore, Mahalanobis distance was used to identify multivariate outliers that could potentially distort parameter estimates. After completing these data-cleaning procedures, 297 valid matched responses were retained for subsequent analyses.

The demographic characteristics of the final sample are described below, while the descriptive statistics and correlations of the coded demographic variables are presented in Table 1. Among the respondents, 55.6% were male and 44.4% were female. The largest age group was 31–35 years old (33.0%), followed by 26–30 years old (29.3%). In terms of education, most respondents held a bachelor’s degree (39.7%), followed by junior college degrees (28.6%) and master’s degrees (19.2%). Regarding work tenure, 37.0% had 5–10 years of work experience, while 27.3% had more than 10 years of experience. In addition, 55.2% of respondents had worked with their current supervisor for 1–3 years, indicating relatively stable supervisor–subordinate relationships. Furthermore, 50.5% of respondents had used generative AI for at least six months, and 48.2% reported using generative AI for more than five hours per week, suggesting substantial experience with AI-assisted work activities.

### 3.2. Measures

This study used established scales that have been widely applied in organizational behavior and information systems research. The original English items were translated into Chinese and checked to ensure semantic equivalence and contextual appropriateness. All measurement items were assessed using a seven-point Likert scale ranging from 1 = “strongly disagree” to 7 = “strongly agree.” For each construct, mean scores were calculated and used in the subsequent analyses. Higher scores indicate higher levels of the corresponding construct. The focal constructs included generative AI use, creative self-efficacy, psychological safety, employee creative behavior, and digital leadership. Detailed information on the measurement items is provided in Table A1.

#### 3.2.1. Generative AI Use

Generative AI use was measured using six items adapted from Burton-Jones and Straub (2006). These items assessed the extent to which employees used generative AI tools in their daily work, including frequency, time spent, task coverage, and functional diversity. Example items include “I frequently use generative AI to complete tasks in my daily work” and “I use generative AI at multiple creative stages, such as clarifying problems, searching information, idea generation, drafting, and polishing.” Higher scores indicate a higher level of generative AI use in work-related tasks. Importantly, the present study does not directly measure the quality, criticality, ethicality, or responsibility of employees’ generative AI use. Accordingly, the empirical claims of this study are limited to the intensity and breadth of work-related generative AI use. Critical evaluation, responsible integration, and potential overreliance are discussed as important qualifications and directions for future research rather than as tested dimensions of the focal construct. The Cronbach’s alpha for this scale was 0.89 in this study.

#### 3.2.2. Creative Self-Efficacy

Creative self-efficacy was measured using the three-item scale developed by Tierney and Farmer (2002). The scale captures employees’ belief in their ability to generate and implement creative ideas. Example items include “I am confident that I can solve problems in a creative way” and “I think I am good at generating creative ideas.” Higher scores indicate stronger confidence in one’s creative capability. The Cronbach’s alpha for this scale was 0.83 in this study.

#### 3.2.3. Psychological Safety

Psychological safety was measured using seven items adapted from Edmondson (1999). Because this study focuses on employees’ individual perceptions rather than team-level aggregated climate, the original team-referenced wording was adapted to refer to employees’ work environment. The scale assesses whether employees feel safe to express ideas, raise questions, ask for help, take interpersonal risks, and use their skills without fear of negative consequences. Example items include “It is safe to take risks in my work environment” and “People in my work environment are able to raise questions and difficult issues.” Three negatively worded items were reverse-coded during data preparation before the composite score was calculated. Higher scores indicate a stronger sense of psychological safety. The Cronbach’s alpha for this scale was 0.90 in this study.

#### 3.2.4. Employee Creative Behavior

Employee creative behavior was measured using six items adapted from Scott and Bruce (1994). These items assess the extent to which employees search for, generate, promote, and implement novel and useful ideas in their work. Example items include “I actively search out new technologies, methods, or ideas” and “I frequently generate new ideas.” Other items capture whether employees promote valuable ideas, secure resources, develop implementation plans, and apply new ideas in their work. Higher scores indicate a higher level of employee creative behavior. The Cronbach’s alpha for this scale was 0.90 in this study.

#### 3.2.5. Digital Leadership

Digital leadership was measured using seven items adapted from Claassen et al. (2021). The scale assesses employees’ perceptions of their direct supervisor’s ability to support digital work practices, encourage the use of digital tools, facilitate digital communication, and promote digital collaboration. Example items include “My direct supervisor encourages the use of digital tools in daily work” and “My direct supervisor demonstrates competence in using digital technology.” Higher scores indicate a higher level of perceived digital leadership. The Cronbach’s alpha for this scale was 0.91 in this study.

#### 3.2.6. Demographic and Background Variables

Several demographic and work-related variables were collected to describe the characteristics of the sample, including gender, age, education level, work tenure, tenure with supervisor, generative AI experience, and weekly generative AI usage. These variables are reported in the descriptive statistics and correlation table.

### 3.3. Data Analysis Strategy

This study used SPSS 27.0 and AMOS 25.0 to analyze the data. First, SPSS was used to conduct descriptive statistics, reliability analysis, and correlation analysis. Cronbach’s alpha coefficients were calculated to assess the internal consistency of the measurement scales. The results showed that the Cronbach’s alpha values of the focal constructs ranged from 0.83 to 0.91, indicating satisfactory reliability.

Second, confirmatory factor analysis (CFA) was conducted using AMOS 25.0 to examine the discriminant validity of the measurement model. The five-factor model included generative AI use, creative self-efficacy, psychological safety, employee creative behavior, and digital leadership. The fit indices showed that the five-factor model achieved good model fit and performed better than alternative models in which several constructs were combined. This result supports the distinctiveness of the core constructs.

Third, parallel mediation analysis was conducted using the PROCESS macro to examine the direct and indirect effects between generative AI use and employee creative behavior through creative self-efficacy and psychological safety. Specifically, the structural model examined whether generative AI use was positively associated with creative self-efficacy and psychological safety, whether these two psychological mechanisms were positively associated with employee creative behavior, and whether generative AI use has a direct effect on employee creative behavior after accounting for the mediators.

Finally, the PROCESS macro was used to examine the mediation, moderation, and moderated mediation effects. Bootstrap procedures with 5000 resamples were applied to estimate indirect effects and confidence intervals. The mediation analysis examined whether creative self-efficacy and psychological safety mediated the relationship between generative AI use and employee creative behavior. The moderation analysis examined whether digital leadership strengthened the effects of generative AI use on creative self-efficacy and psychological safety. The moderated mediation analysis further tested whether the indirect effects of generative AI use on employee creative behavior through creative self-efficacy and psychological safety varied across different levels of digital leadership.

## 4. Results

### 4.1. Descriptive Statistics and Correlations

The descriptive statistics, reliability coefficients, and correlations among the study variables are presented in Table 1. The results show that the Cronbach’s alpha values of the focal constructs ranged from 0.83 to 0.91, exceeding the commonly recommended threshold of 0.70 and indicating good internal consistency. Specifically, generative AI use, creative self-efficacy, psychological safety, employee creative behavior, and digital leadership all demonstrated satisfactory reliability.

The correlation results further show that the core variables were positively and significantly related to one another. Generative AI use was positively correlated with creative self-efficacy, psychological safety, employee creative behavior, and digital leadership. Creative self-efficacy and psychological safety were also positively associated with employee creative behavior. These results are generally consistent with the theoretical expectations and provide preliminary support for the proposed relationships. In addition, the correlation coefficients did not indicate serious multicollinearity among the focal variables.

### 4.2. Confirmatory Factor Analysis

To examine the discriminant validity of the key constructs, this study conducted confirmatory factor analysis using AMOS 25.0. The results are reported in Table 2. The proposed five-factor model, including generative AI use, creative self-efficacy, psychological safety, digital leadership, and employee creative behavior, showed good model fit: χ^2^ = 406.530, df = 367, χ^2^/df = 1.108, RMSEA = 0.019, SRMR = 0.034, CFI = 0.981, and TLI = 0.990 (Hu & Bentler, 1999).

The five-factor model was further compared with several alternative models, including four-factor, three-factor, two-factor, and single-factor models. The results show that the five-factor model fit the data substantially better than all competing models. As different constructs were gradually combined, model fit deteriorated, as reflected by lower CFI and TLI values and higher RMSEA and SRMR values. These findings indicate that the five focal constructs are empirically distinct and provide support for the discriminant validity of the measurement model.

### 4.3. Measurement Model Results

This study further assessed the reliability, convergent validity, and discriminant validity of the measurement model. As shown in Table 3, all standardized factor loadings were above 0.70, ranging from 0.731 to 0.809, indicating adequate indicator reliability. The composite reliability values ranged from 0.83 to 0.91, exceeding the recommended threshold of 0.70 (Bagozzi & Yi, 1988). In addition, the average variance extracted values ranged from 0.57 to 0.61, all above the recommended threshold of 0.50 (Bagozzi & Yi, 1988; Fornell & Larcker, 1981). These results suggest that the measurement model has good internal consistency and convergent validity.

Discriminant validity was assessed using the Fornell–Larcker criterion (Fornell & Larcker, 1981). The square roots of AVE were AGU = 0.763, CSE = 0.783, PS = 0.758, ECB = 0.768, and DL = 0.762. These values exceeded the corresponding inter-construct correlations, supporting discriminant validity. Overall, the results support the reliability, convergent validity, and discriminant validity of the measurement model.

### 4.4. Parallel Mediation Analysis

To provide a coherent examination of the proposed mediation model, a parallel mediation analysis was conducted to estimate the total effect, direct effect, two specific indirect effects, and total indirect effect between generative AI use and employee creative behavior. Creative self-efficacy and psychological safety were simultaneously included as parallel mediators, and bootstrap confidence intervals were calculated based on 5000 resamples. The results are presented in Table 4. The total effect between generative AI use and employee creative behavior was positive and significant (B = 0.3627, SE = 0.0546, t = 6.648, *p* < 0.001, 95% CI [0.2553, 0.4701]), supporting H1. After creative self-efficacy and psychological safety were simultaneously included in the model, the direct effect between generative AI use and employee creative behavior remained significant (B = 0.2600, SE = 0.0559, t = 4.649, *p* < 0.001, 95% CI [0.1499, 0.3700]). The specific indirect effect through creative self-efficacy was significant (Effect = 0.0450, SE = 0.0166, 95% bootstrap CI [0.0166, 0.0807]), supporting H2. The specific indirect effect through psychological safety was also significant (Effect = 0.0577, SE = 0.0202, 95% bootstrap CI [0.0210, 0.1006]), supporting H3. The total indirect effect through the two mediators was 0.1027 (SE = 0.0256, 95% bootstrap CI [0.0569, 0.1586]). Because the direct effect remained significant after both mediators were simultaneously included, the results indicate partial mediation. Specifically, generative AI use was associated with employee creative behavior both directly and indirectly through creative self-efficacy and psychological safety.

### 4.5. Moderation and Moderated Mediation Analysis

To examine the moderating role of digital leadership, this study used PROCESS Model 7 with 5000 bootstrap samples (Hayes, 2017; Preacher et al., 2007). The results are reported in Table 5.

**Figure 2:**
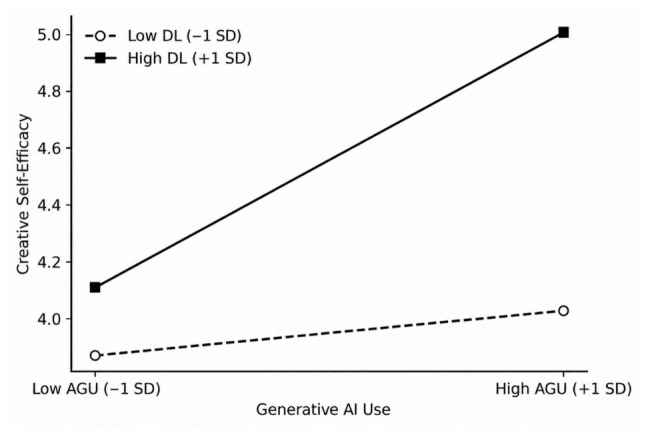
Moderating effect of digital leadership on the relationship between generative AI use and creative self-efficacy.

As shown in Panel A of Table 5, the interaction between generative AI use and digital leadership had a significant positive effect on creative self-efficacy (Effect = 0.1104, *p* < 0.05). This result indicates that digital leadership strengthened the positive relationship between generative AI use and creative self-efficacy. Thus, H4 was supported. As illustrated in Figure 2, the positive relationship between generative AI use and creative self-efficacy was stronger when digital leadership was high. This pattern is consistent with employees perceiving clearer technological guidance, stronger digital support, and greater encouragement for responsible experimentation under high digital leadership.

**Figure 3:**
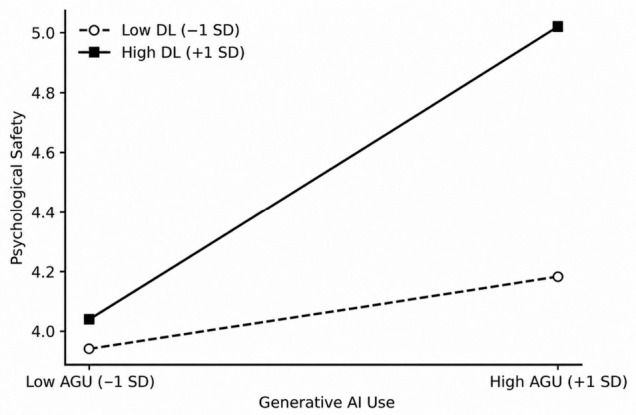
Moderating effect of digital leadership on the relationship between generative AI use and psychological safety.

As shown in Panel B of Table 5, the interaction between generative AI use and digital leadership had a significant positive effect on psychological safety (Effect = 0.1105, *p* < 0.01). This result indicates that digital leadership strengthened the positive relationship between generative AI use and psychological safety. Thus, H5 was supported. As illustrated in Figure 3, this positive relationship was stronger when digital leadership was high than when it was low. Specifically, under high digital leadership, employees were more likely to perceive AI-supported creative work as acceptable and supported, which was associated with lower uncertainty and interpersonal risk when expressing ideas, sharing AI-assisted outputs, and engaging in experimentation.

Panel B of Table 5 reports the conditional indirect effects at different levels of digital leadership. For the creative self-efficacy pathway, the indirect effect between generative AI use and employee creative behavior was not significant when digital leadership was low (Effect = 0.0092, 95% CI [−0.0215, 0.0380]). However, the indirect effect became significant at the mean level of digital leadership (Effect = 0.0367, 95% CI [0.0130, 0.0659]) and at the high level of digital leadership (Effect = 0.0729, 95% CI [0.0292, 0.1266]). These results indicate that digital leadership strengthened the indirect effect of generative AI use on employee creative behavior through creative self-efficacy.

Similarly, for the psychological safety pathway, the indirect effect of generative AI use on employee creative behavior was not significant when digital leadership was low (Effect = 0.0181, 95% CI [−0.0193, 0.0559]). However, the indirect effect became significant at the mean level of digital leadership (Effect = 0.0502, 95% CI [0.0199, 0.0888]) and at the high level of digital leadership (Effect = 0.0926, 95% CI [0.0409, 0.1572]). These findings suggest that digital leadership also strengthened the indirect effect of generative AI use on employee creative behavior through psychological safety.

Finally, Panel C of Table 5 reports the indices of moderated mediation. The index for the creative self-efficacy pathway was 0.0214, with a 95% confidence interval of [0.0045, 0.0435]. The index for the psychological safety pathway was 0.0250, with a 95% confidence interval of [0.0059, 0.0503]. Since neither confidence interval included zero, the moderated mediation results further supported the proposed moderated dual-mediation model. Taken together, these results indicate that the conditional indirect effects between generative AI use and employee creative behavior through both creative self-efficacy and psychological safety were stronger at higher levels of digital leadership.

**Figure 4:**
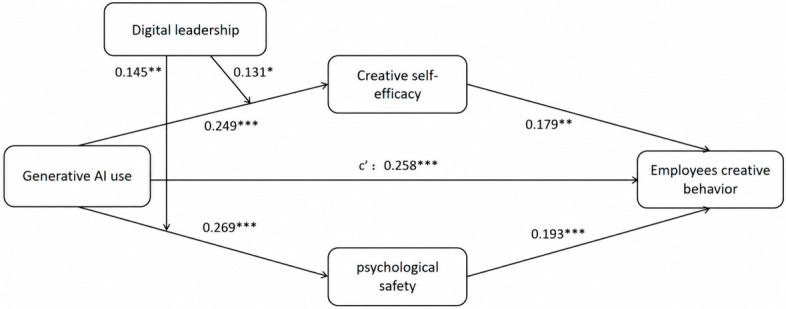
Summary of the mediation, moderation, and moderated mediation results. Note: Standardized coefficients are shown. The mediation-path coefficients and the AGU–ECB direct effect are based on the parallel mediation analysis, whereas the interaction coefficients are based on the moderation analyses. The total standardized effect of AGU on ECB was β = 0.359, *p* < 0.001. * *p* < 0.05, ** *p* < 0.01, *** *p* < 0.001.

To provide a visual overview of the empirical findings, Figure 4 summarizes the direct, mediating, and moderating relationships examined in this study. The AGU–ECB path represents the direct effect after creative self-efficacy and psychological safety were simultaneously included in the parallel mediation model. The total effect, the direct effect, the specific indirect effects, and the total indirect effect are reported in Table 4. The moderation effects, conditional indirect effects, and indices of moderated mediation are reported in Table 5. Overall, the findings indicate that generative AI use is associated with employee creative behavior both directly and indirectly through creative self-efficacy and psychological safety, and that the two indirect effects are stronger at higher levels of digital leadership.

## 5. Discussion

This study investigated how generative AI use is associated with employee creative behavior through two psychological pathways: creative self-efficacy and psychological safety, and further examined the moderating role of digital leadership. The findings show that generative AI use is positively related to employee creative behavior, and that this relationship is explained by both creative self-efficacy and psychological safety. These results are consistent with prior studies suggesting that generative AI can support knowledge work, task efficiency, and creative activities by providing information, alternative perspectives, and idea-generation support (Dell’Acqua et al., 2023; Füller et al., 2022; Jia et al., 2024; Mariani & Dwivedi, 2024; Noy & Zhang, 2023; Verganti et al., 2020; Wang et al., 2026). However, this study extends previous research by shifting the focus from productivity, task performance, or general creative outputs to employee creative behavior in organizational settings. In addition, the mediating effects of creative self-efficacy and psychological safety indicate that generative AI use is associated with employee creative behavior through both stronger confidence in employees’ creative capabilities and lower perceived interpersonal risks during AI-assisted creative work. This finding is consistent with Social Cognitive Theory (Bandura, 1986, 1997), creative self-efficacy research (Oldham & Cummings, 1996; Tierney & Farmer, 2002, 2011), and psychological safety research (Edmondson, 1999; Frazier et al., 2017; Hülsheger et al., 2009), while further extending these perspectives to the context of AI-supported work. Finally, digital leadership strengthens these psychological pathways, which is consistent with prior research on digital leadership and digital transformation (Avolio et al., 2014; Kane et al., 2019; Klus & Müller, 2021; Zeike et al., 2019). More importantly, the findings suggest that digital leadership does not merely support innovation in general; higher digital leadership is associated with stronger relationships between generative AI use and both creative confidence and psychological safety, which are, in turn, associated with employee creative behavior.

Taken together, these findings offer a more comprehensive understanding of how technological resources are linked to employee creative behavior through psychological mechanisms and supportive digital leadership.

### 5.1. Theoretical Implications

First, this study contributes to the emerging literature on generative AI and employee creativity by clarifying how generative AI use is associated with employee creative behavior in organizational settings. Rather than treating generative AI as a tool that automatically produces creative outcomes, the findings suggest that the effect between generative AI use and employee creative behavior is partly explained by employees’ psychological responses. This is particularly important because generative AI differs from conventional digital tools by offering interactive idea generation, real-time feedback, iterative refinement, and human–AI co-creation support during creative work (Jia et al., 2024; Wang et al., 2026). By identifying creative self-efficacy and psychological safety as two complementary mechanisms, this study provides a more nuanced explanation of how generative AI use is linked to employee creative behavior.

Second, this study advances knowledge regarding the psychological pathways linking generative AI use to employee creative behavior. Specifically, the findings identify two distinct yet complementary pathways: a capability-enhancement pathway through creative self-efficacy and a risk-reduction pathway through psychological safety. The creative self-efficacy pathway suggests that AI-supported experiences strengthen employees’ confidence in their ability to generate novel and useful ideas, thereby enhancing their willingness to engage in creative activities (Bandura, 1997; Tierney & Farmer, 2002). In contrast, the psychological safety pathway highlights how generative AI can reduce perceived interpersonal risks associated with creative expression and experimentation, making employees more willing to share, refine, and implement innovative ideas (Edmondson, 1999; Frazier et al., 2017). By simultaneously examining these two pathways, this study provides a more comprehensive explanation of how AI-assisted work experiences are linked to employee creative behavior through both capability perception and interpersonal risk perception. More importantly, the findings suggest that generative AI use is linked to employee creative behavior through both stronger capability perceptions and lower perceived social and interpersonal risks associated with innovation.

Third, this study extends Psychological Safety Theory to the context of AI-assisted work. Previous research has primarily examined psychological safety in relation to leadership behavior, interpersonal relationships, team learning, and organizational communication (Edmondson & Lei, 2014). However, the increasing integration of generative AI into workplace activities introduces new forms of uncertainty and social risk. Employees may worry about being judged as overly dependent on AI, lacking originality, or relying excessively on machine-generated content. Consequently, psychological safety becomes increasingly important in helping employees manage the interpersonal and identity-related risks associated with AI-assisted work. By identifying psychological safety as a key mediating mechanism, this study expands the theoretical boundaries of psychological safety research and demonstrates its continued relevance in digitally enabled and AI-supported work environments.

Finally, this study highlights the important role of digital leadership as a contextual condition that clarifies how digital leadership conditions the effects between generative AI use and employees’ psychological responses (Zhu et al., 2022). The findings suggest that the benefits of generative AI are not automatically realized simply through access to technology. Instead, leaders play a critical role in shaping how employees interpret, utilize, and integrate AI into their work processes. By providing technological guidance, resource support, and a climate that encourages responsible experimentation, digital leadership strengthens both the capability-enhancement and risk-reduction mechanisms identified in this study. This finding enriches our understanding of leadership in digital transformation contexts and suggests that supportive digital leadership may strengthen the effect between generative AI use and employee creative behavior.

### 5.2. Practical Implications

For organizational policy, organizations should position generative AI as a tool for creative exploration rather than merely as a shortcut for routine tasks. Employees may use generative AI to generate alternative perspectives, compare possible solutions, and refine early-stage ideas. At the same time, organizations should provide clear guidelines on appropriate AI use, including issues related to accuracy, data security, authorship, and ethical responsibility. Without such guidelines, employees may either avoid using AI because of uncertainty or rely on it too heavily without critical evaluation (Bankins et al., 2024; Dwivedi et al., 2023).

For employee training, AI training should go beyond basic tool operation and emphasize how generative AI can support creative problem solving in real work tasks. Training can include prompt design, idea comparison, output evaluation, and revision of AI-generated content. Managers can also provide feedback on employees’ AI-assisted ideas, especially when employees use AI in thoughtful and original ways. Such experiences can help employees develop confidence in their ability to use AI as part of the creative process.

For responsible AI use, organizations should create a psychologically safe environment for AI-supported experimentation. Employees may hesitate to share AI-assisted ideas when they worry about being judged as less competent, less original, or overly dependent on AI. Managers should therefore encourage open discussion about how AI is used, what its limitations are, and how AI-generated outputs can be improved. It is also useful to normalize trial and error in AI-supported work, because not every AI output will be accurate, useful, or creative (Dwivedi et al., 2023). When employees feel that imperfect attempts can be discussed rather than punished, they are more likely to experiment with AI and transform preliminary outputs into creative solutions.

For leadership, digital leaders should clarify the purpose, value, and boundaries of generative AI use. In the case of generative AI, leaders should provide clear expectations, encourage responsible use, and support employees when they explore new ways of working. Leaders can also model appropriate AI use by showing how AI outputs should be evaluated, revised, and combined with human judgment. Such leadership practices can strengthen employees’ creative confidence and reduce interpersonal uncertainty, thereby making it more likely that generative AI use is associated with employee creative behavior.

### 5.3. Boundary Conditions and Potential Risks of Generative AI Use

The findings should also be interpreted in light of the potentially double-edged nature of generative AI use. Although this study examined only positive psychological pathways and did not empirically test potential negative effects, prior research suggests that the creative value of generative AI depends on employees’ critical evaluation and responsible integration of AI-generated content (Dwivedi et al., 2023; Jarrahi, 2018; Shan et al., 2026). Generative AI may strengthen creative self-efficacy by helping employees compare and refine ideas, but excessive reliance may weaken independent problem-solving and creative confidence (Lee & Moray, 1994; Parasuraman & Riley, 1997). It may also help employees refine ideas before sharing them, whereas inaccurate or standardized outputs may raise concerns about originality and contribute to creative homogenization (Doshi & Hauser, 2024). These potential risks are not empirical findings of this study but boundary considerations for interpreting the results. Therefore, organizations and digital leaders should guide employees to use generative AI as a supportive tool while maintaining critical thinking, professional judgment, and responsibility for final outputs (Bankins et al., 2024; Jarrahi, 2018).

### 5.4. Limitations and Future Research

Several limitations should be acknowledged. First, although this study used a three-wave time-lagged design, all variables were measured through self-reported questionnaires. Therefore, the findings should be interpreted as time-ordered effects rather than definitive causal evidence. In addition, the measure of generative AI use mainly captures usage intensity and breadth, rather than the quality, criticality, responsibility, or possible overreliance involved in AI use. Future research could use experimental, longitudinal, multi-source, or experience-sampling designs and develop more fine-grained measures of generative AI use.

Second, this study focused mainly on the positive role of generative AI use. However, the relationship between generative AI use and creativity may also have negative sides. For example, employees may become overly dependent on AI-generated suggestions, rely on similar prompts, or converge toward standardized solutions. Such patterns may reduce originality and diversity in creative outputs. Future research could adopt a double-edged-sword perspective to examine when generative AI use is positively associated with creativity and when it may be associated with creative homogenization, cognitive dependence, or reduced independent thinking.

Third, the study examined creative self-efficacy and psychological safety as two mediating mechanisms, but other mechanisms may also be relevant. For example, intrinsic motivation, AI trust, perceived autonomy, learning behavior, work engagement, or cognitive load may further explain how generative AI use is associated with employee creativity. Future studies could compare these mechanisms to determine which psychological processes are most important under different task conditions.

Finally, this study focused on digital leadership as a moderator. Other contextual factors may also influence whether generative AI use is associated with creative outcomes. Future research could examine organizational AI policies, innovation climate, ethical AI governance, task complexity, AI literacy, or industry differences. Such work would help clarify the conditions under which generative AI is most likely to support employee creativity in real organizational settings. In addition, the sample was drawn from employees working in highly digitalized enterprises in China. Cultural norms, organizational digital maturity, leadership expectations, and AI adoption policies may influence how employees use generative AI and how they perceive psychological safety. Therefore, the findings should be generalized to other countries, industries, and work cultures with caution. Future studies could test the model in different cultural and sectoral contexts.

## 6. Conclusions

This study examined how generative AI use is associated with employee creative behavior by focusing on the mediating roles of creative self-efficacy and psychological safety and the moderating role of digital leadership. Drawing on social cognitive theory and psychological safety theory, this study developed and tested a moderated dual-mediation model using survey data from 297 employees. The findings show that generative AI use is positively associated with employee creative behavior. In addition, creative self-efficacy and psychological safety both serve as important psychological mechanisms through which generative AI use is associated with creative behavior. These results suggest that generative AI may support employee creativity not only by providing direct cognitive support, but also through employees’ creative confidence and perceived interpersonal safety.

Furthermore, this study found that digital leadership serves as an important boundary condition in this process. Specifically, the positive effects between generative AI use and both creative self-efficacy and psychological safety were stronger at higher levels of digital leadership, and the corresponding conditional indirect effects with employee creative behavior were also stronger. These findings indicate that the creative value of generative AI is more likely to be realized when leaders provide clear digital guidance, technological support, and an environment that encourages responsible experimentation.

Overall, this study provides a systematic explanation of how generative AI use is linked to employee creative behavior in AI-supported workplaces. It highlights the importance of integrating technological resources, employee psychological mechanisms, and leadership context when understanding creativity in AI-supported workplaces. The findings also offer practical guidance for organizations seeking to use generative AI not only to improve efficiency, but also to encourage employee creativity and support innovation in the digital era.

## Figures and Tables

**Table 1:** Descriptive Statistics, Reliability, and Correlations.

	1	2	3	4	5	6	7	8	9	10	11	12
1. Gender	—											
2. Age	−0.038	—										
3. Education	−0.046	0.168 **	—									
4. Work tenure	0.023	0.879 ***	−0.115 *	—								
5. Tenure with supervisor	0.061	0.233 ***	−0.029	0.343 ***	—							
6. AI experience	0.029	0.103	−0.059	0.109	0.059	—						
7. Weekly AI usage	−0.093	0.063	0.003	0.039	0.006	0.565 ***	—					
8. AGU	−0.060	−0.023	−0.027	−0.025	−0.092	0.403 ***	0.481 ***	(0.89)				
9. CSE	−0.023	−0.058	−0.041	−0.062	0.015	0.292 ***	0.319 ***	0.249 ***	(0.83)			
10. PS	0.020	0.097	0.042	0.073	0.077	0.447 ***	0.466 ***	0.296 ***	0.218 ***	(0.90)		
11. ECB	0.019	0.021	0.027	0.014	0.040	0.482 ***	0.458 ***	0.359 ***	0.285 ***	0.309 ***	(0.90)	
12. DL	0.009	−0.021	−0.103	0.035	−0.022	0.370 ***	0.405 ***	0.206 ***	0.270 ***	0.249 ***	0.274 ***	(0.91)
M	1.45	3.083	2.88	3.773	2.907	3.373	3.313	4.356	4.292	4.334	4.337	4.27
SD	0.498	1.096	0.998	1.058	0.726	1.291	1.319	1.295	1.41	1.279	1.307	1.292

Note(s): AGU = generative AI use; CSE = creative self-efficacy; PS = psychological safety; ECB = employee creative behavior; DL = digital leadership. * *p* < 0.05, ** *p* < 0.01, *** *p* < 0.001.

**Table 2:** Comparison of measurement models.

	χ^2^	df	χ^2^/df	RMSEA	SRMR	CFI	TLI
Reference			<3	<0.08	<0.08	>0.9	>0.9
Five-factor model	406.530	367.000	1.108	0.019	0.034	0.981	0.990
Four-factor model	1254.456	371.000	3.381	0.089	0.148	0.805	0.787
Three-factor model	1587.176	374.000	4.244	0.104	0.197	0.733	0.710
Two-factor model	2353.180	376.000	6.259	0.133	0.193	0.564	0.529
Single-factor model	3060.370	377.000	8.117	0.154	0.233	0.408	0.363

Note: Five-factor model: AGU, CSE, PS, ECB, DL; Four-factor model: AGU, CSE, PS, ECB + DL; Three-factor model: AGU, CSE + PS, ECB + DL; Two-factor model: AGU + CSE + PS, ECB + DL; Single-factor model: AGU + CSE + PS + ECB + DL.

**Table 3:** Measurement Model Results: Reliability and Convergent Validity.

Construct	Item	Loading	CR	AVE
AGU	AGU1	0.750	0.89	0.58
	AGU2	0.757		
	AGU3	0.770		
	AGU4	0.760		
	AGU5	0.771		
	AGU6	0.770		
CSE	CSE1	0.809	0.83	0.61
	CSE2	0.787		
	CSE3	0.753		
PS	PS1	0.770	0.90	0.57
	PS2	0.767		
	PS3	0.797		
	PS4	0.735		
	PS5	0.731		
	PS6	0.742		
	PS7	0.759		
ECB	ECB1	0.762	0.90	0.59
	ECB2	0.775		
	ECB3	0.791		
	ECB4	0.748		
	ECB5	0.766		
	ECB6	0.765		
DL	DL1	0.782	0.91	0.58
	DL2	0.767		
	DL3	0.770		
	DL4	0.765		
	DL5	0.743		
	DL6	0.749		
	DL7	0.760		

**Table 4:** Total, Direct, and Indirect Effects of the Parallel Mediation Model.

Path	Effect	SE	t	*p*	95% CI	Result
Total effect: AGU → ECB, c	0.3627	0.0546	6.648	***	[0.2553, 0.4701]	Supported (H1)
Direct effect: AGU → ECB, c′	0.2600	0.0559	4.649	***	[0.1499, 0.3700]	Significant
AGU → CSE → ECB	0.0450	0.0166	—	—	[0.0166, 0.0807]	Supported (H2)
AGU → PS → ECB	0.0577	0.0202	—	—	[0.0210, 0.1006]	Supported (H3)
Total indirect effect	0.1027	0.0256	—	—	[0.0569, 0.1586]	Significant

Note: Bootstrap = 5000. CI = 95%. *** *p* < 0.001.

**Table 5:** Moderation and Moderated Mediation Results.

**Panel A. Moderation Effects**					
**Outcome**	**Predictor**	**Effect**	**SE**	**t**	***p***
CSE	AGU	0.2035	0.0608	3.35	***
	DL	0.2361	0.0608	3.89	***
	AGU × DL	0.1104	0.0466	2.37	*
PS	AGU	0.2359	0.0546	4.32	***
	DL	0.1818	0.0546	3.33	**
	AGU × DL	0.1105	0.0419	2.64	**
**Panel B. Conditional Indirect Effects**					
**Path**		**DL Level**	**Effect**	**BootSE**	**95% CI**
AGU → CSE → ECB		Low	0.0092	0.0149	[−0.0215, 0.0380]
		Mean	0.0367	0.0137	[0.0130, 0.0659]
		High	0.0729	0.0255	[0.0292, 0.1266]
AGU → PS → ECB		Low	0.0181	0.0191	[−0.0193, 0.0559]
		Mean	0.0502	0.0177	[0.0199, 0.0888]
		High	0.0926	0.0301	[0.0409, 0.1572]
**Panel C. Indices of Moderated Mediation**					
**Path**			**Effect**	**BootSE**	**95% CI**
AGU → CSE → ECB			0.0214	0.0101	[0.0045, 0.0435]
AGU → PS → ECB			0.0250	0.0115	[0.0059, 0.0503]

Note(s): Bootstrap = 5000. CI = 95%. * *p* < 0.05, ** *p* < 0.01, *** *p* < 0.001.

## Data Availability

The data supporting this study’s findings are available from the corresponding author upon reasonable request.

## Appendix A

**Table A1:** Measurement.

Variables	Title Content	Scale Source
Generative AI Use	1. I frequently use generative AI to complete tasks in my daily work.	Burton-Jones and Straub (2006)
	2. In a typical work week, I interact with generative AI many times.	
	3. In a typical work/study week, I spend a considerable amount of time using generative AI.	
	4. A large proportion of my task time is spent interacting with generative AI.	
	5. I use generative AI at multiple creative stages (e.g., clarifying problems, searching information, idea generation, drafting, polishing).	
	6. I use multiple functions of generative AI (e.g., multi-turn prompting, code/data analysis, outlines, summaries, etc.).	
Creative Self-Efficacy	1. I am confident that I can solve problems in a creative way.	Tierney and Farmer (2002)
	2. I think I am good at generating creative ideas.	
	3. I believe I can successfully implement creative ideas.	
Employee creative behavior	1. I actively search out new technologies, methods, or ideas.	Scott and Bruce (1994)
	2. I frequently generate new ideas.	
	3. I promote and champion valuable new ideas.	
	4. I coordinate or secure resources to implement new ideas.	
	5. I develop plans for implementing new ideas.	
	6. I actually try out and apply new ideas in my work.	
Digital Leadership	1. My direct supervisor encourages the use of digital tools in daily work.	Claassen et al. (2021)
	2. My direct supervisor supports employees in dealing with issues related to digital technology.	
	3. My direct supervisor promotes digital collaboration and knowledge sharing.	
	4. My direct supervisor ensures smooth and effective digital communication in the work environment.	
	5. My direct supervisor provides clear information through digital channels.	
	6. My direct supervisor demonstrates competence in using digital technology.	
	7. My direct supervisor actively promotes digital work processes.	
Psychological Safety	1. If I make a mistake in my work environment, it is usually held against me. (reverse)	Edmondson (1999)
	2. People in my work environment are able to raise questions and difficult issues.	
	3. People in my work environment sometimes reject others for being different. (reverse)	
	4. It is safe to take risks in my work environment.	
	5. It is difficult to ask others in my work environment for help. (reverse)	
	6. People in my work environment do not deliberately act in a way that undermines my efforts.	
	7. In my work environment, my unique skills and talents are valued and utilized.	
